# Implementing trauma-informed practice across services to support people experiencing multiple disadvantage: a mixed method study

**DOI:** 10.1186/s12913-025-13339-8

**Published:** 2025-10-01

**Authors:** Michelle Farr, Emily Eyles, Tracey Stone, Maria Theresa Redaniel, Thomas Traub, Jason Burrowes, Rebecca Halsley, Katherine Williams, Aileen Edwards, Sabi Redwood

**Affiliations:** 1 The National Institute for Health and Care Research Applied Research Collaboration West (NIHR ARC West) at University Hospitals Bristol and Weston NHS Foundation Trust, Bristol, UK; 2 Population Health Sciences, Bristol Medical School, University of Bristol, Bristol, UK; 3 National Cancer Registry Ireland, Cork, Ireland; 4 Independent Futures, Second Step, Bristol, UK; 5 Changing Futures Bristol, Bristol City Council, Bristol, UK; 6 Second Step, Bristol, UK

**Keywords:** Trauma-informed, Multiple disadvantage, Homelessness, Addiction, Mental health, Violence or abuse, Co-production

## Abstract

**Background:**

People facing multiple disadvantage have often experienced extensive trauma. Changing Futures Bristol was part of a national programme to improve outcomes for people who face multiple disadvantage, such as combinations of homelessness, substance misuse, mental ill-health, domestic violence and abuse or contact with the criminal justice system. Aims were to understand how services could be improved, with more trauma-informed approaches at individual, service and system levels. An in-depth mixed method evaluation of Changing Futures Bristol examined how trauma-informed approaches were implemented and linked across services supporting people experiencing multiple disadvantage.

**Methods:**

The study followed a participatory action research approach, involving research conducted in collaboration with people who have experienced multiple disadvantage, and staff partners. Collaborators actively contributed to securing funding, research design, data analysis, and write-up. A staff survey was conducted using existing measures and some tailored questions, to assess perceptions of trauma-informed approaches, equality, diversity and inclusion, and co-production. One hundred and seventeen staff responded, with 30 staff completing the survey again after one year to track any changes. Twenty-three staff members were interviewed. Qualitative data were analysed thematically, guided by trauma-informed principles and implementation domains.

**Results:**

Movement toward more trauma-informed approaches was detected, although these changes were not found to be statistically significant after one year. Barriers included short-term funding and commissioning cycles and difficulties in staff retention, due to short-term contracts, vicarious trauma, stress and pressures of the job. Managers had to hold contradicting drivers to deliver targets and manage finances whilst creating space for relational support and trauma-informed practice. To create psychological safety, staff needed to feel trust and transparency. 73% of staff reported lived experience of at least one domain of multiple disadvantage or trauma. Support for staff is needed at all levels of the organisation.

**Conclusions:**

A long-term, collaborative, and trauma-informed approach is needed at all levels, including leaders, managers, policymakers, and central government. Government and public service reforms that focus on cross-sector collaboration and devolution of power will support trauma-informed practices. Stable, long-term funding and planning will help create a motivated, skilled workforce that can build on existing good practice.

**Supplementary Information:**

The online version contains supplementary material available at 10.1186/s12913-025-13339-8.

## Background

People who experience multiple disadvantage (MD) (defined as people who have faced combinations of homelessness, drug or alcohol problems, mental ill health, domestic violence and abuse or contact with the criminal justice system) often have had backgrounds of adverse childhood experiences, adversity or trauma [1–3]. Trauma comes from events that are physically or emotionally harmful or life threatening [4, 5]. One study found that 85% of people experiencing multiple disadvantage had experienced traumatic experiences in childhood [1]. Experiences of multiple disadvantage can be traumatic in themselves and can compound earlier adverse childhood experiences [2] and cause considerable ill-health, frailty and premature mortality [6, 7].

It has been highlighted that trauma-informed (TI) practice can be helpful for individuals experiencing multiple disadvantage and for staff providing support [8]. To be able to implement TI practice, change is required at multiple levels of an organisation, with sustained effort over time [9], and a reconstitution of organisational systems to embed values such as emotional intelligence, democracy, open communication and social responsibility [10]. The Substance Abuse and Mental Health Services Administration (SAMHSA) [4] build on previous TI work [9, 10] to identify ten implementation domains at multiples levels of an organisation, that can be aligned with the six key TI principles, to undertake organisational change management to establish TI care (Table 1).

**Table 1:** Six TI principles and 10 implementation domains

**Trauma-informed principles** [4]	**Details** [4]
Cultural, Historical and Gender issues	Tackling historic, gender and cultural issues to avoid biases and stereotypes and recognise historical trauma. Responding to individual’s needs from different racial, ethnic, religious and cultural backgrounds and different genders [4].
Peer Support	Peer support involves people with lived experience of trauma supporting each other directly, with relationships based on mutuality and collaboration [11].
Trustworthiness and Transparency	Transparency and clarity in how decisions are made, in order to develop and maintain trust [4].
Collaboration	Partnership working and co-production that recognises that everyone working together has equal value. Work is done to identify and reduce unequal power dynamics [4]*.*
Empowerment and Choice	Recognising the strengths and resilience of people who’ve experienced trauma and the potential to heal. Supporting shared decision making [4].
Safety	Environments and cultures are physically and emotionally safe where all members feel supported. Interpersonal interactions encourage a sense of safety [4].
**TI Implementation Domains** [4]	**Details** [4]
Governance & Leadership	Leadership is invested in sustaining a TI approach. Support and investment in TI practice is included at all levels of an organisation and ideally within policy and commissioning through multiple systems.
Policy	Written policies commit to and promote TI practice as an essential part of organisation mission. Wider system policies that affect the context within which organisations operate also include TI practices.
Physical environment	The physical environment promotes a sense of safety and collaboration, with safe and inviting spaces that are not a risk to physical or psychological safety.
Engagement & involvement	People who have experienced trauma/ people receiving services have significant involvement, voice and choice within all organisational areas, including policy, service design, delivery, peer support, evaluation.
Cross sector collaboration	Collaboration across sectors is based on a shared understanding of trauma, so that when people need to access services across multiple sectors, this is joined up and done in a trauma-informed way.
Screening, assessment, treatment (support) services	Practitioners use and are trained in trauma-informed, evidence-based interventions, that support people who have experienced trauma in a person-centred, strengths-based way.
Training & workforce development	Training in trauma-informed approaches is key, human resources incorporate TI practice in recruitment, supervision, and staff support. Additional support for vicarious trauma is available.
Progress, monitoring & quality assurance	Assessment and monitoring of trauma-informed principles within practice.
Financing	Financing includes resources for trauma training, development of appropriate and safe spaces, peer support, provision of trauma treatment and support for recovery, and TI collaborations.
Evaluation	Evaluation is conducted in a way that is trauma-informed and uses appropriate TI methods.

Implementing TI approaches have shown positive results within mental health services, child welfare and the criminal justice system, through strong leadership and organisational buy-in [12]. TI approaches can be an effective way of working with people experiencing multiple disadvantage through on-going personalised support and trusting relationships with support staff, enabled by low caseloads [13]. However, TI implementation can be affected by system values, policies, governance, business models, organisational culture, buy-in from staff and sustainable funding [14]. Reviews of TI approaches advocate a greater focus on marginalised populations, and cultural and gender sensitive approaches [15].

Intersectionality is a useful framework to understand how different forms of discrimination impact trauma and multiple disadvantage, with discriminatory practices compounding together to create further disadvantage [16]. Routes into and experiences of multiple disadvantage can be highly gendered, with women experiencing high levels of sexual violence and domestic abuse. Homelessness can also be more hidden [17]. Racism and racial trauma can compound disadvantage, from experiences of harm, threat, prejudice and humiliation due to racial discrimination [18]. Racial trauma is understood to differ from complex trauma in three core ways: its origin within a racist ideology of White supremacy; its constancy beyond childhood as an inescapable element within institutions and social structures; and internalization of dominant groups’ values, attitudes and ideologies [18].

Much theoretical, empirical and clinical work focuses on trauma within individuals, however it is vital to understand how institutions, cultures, and systems impact on trauma [19]. Health, welfare and criminal justice systems, and lack of appropriate housing may adversely impact and cause further trauma and multiple disadvantage [20]. Experiencing homelessness, violence or abuse are inherently traumatic experiences which can compound earlier adverse childhood experiences and traumas [2, 21]. Involvement in the criminal justice system can exacerbate previous harms [22]. Whilst the origins of trauma-informed approaches highlight the importance of incorporating trauma principles into organisational cultures [4], this work needs to take place at a national policy level and over the wider public service system [23]. Whilst multiple disadvantage by its very nature means that a cross-sector collaborative approach is a necessity, organisational silos including from national government department levels down to a local level can increase the risk of duplication, and produce gaps in specialist support [24, 25]. At a national level there can be a tendency to focus on new announcements and policy design, rather than building on good practice to improve the quality of existing provision [24, 25]. Systems change can be a challenge at all levels [24, 25]. There are also challenges in evaluating the implementation and effectiveness of TI approaches, due to different definitions and the need for further research on TI measurement [26].

### The Changing Futures programme and Bristol case site

The Changing Futures programme aimed to support innovative approaches for people who have experienced multiple disadvantage (MD) through a £77 million UK government and National Lottery Community Fund initiative running in 15 areas across England from 2021 to 2025 [3], with a further £15 million extension until April 2026 [27]. Previous to Changing Futures, the Fulfilling Lives programme was funded through the UK National Lottery to address the needs of people with multiple disadvantage. The national evaluation of Fulfilling Lives highlighted that complex systems change was slow, and that the eight-year funding period without hard targets enabled flexibility, the ability to take risks, and build buy-in and trust between staff, services, partners and commissioners [28]. Our study moves beyond Fulfilling Lives and national Changing Futures evaluations by providing an in-depth analysis of staff perspectives and the challenges experienced by leaders in implementing trauma-informed approaches at a systems level, plus new data on staff lived experience.

Changing Futures Bristol (CFB) was initially set up as a three-year programme (July 2021- March 2024) with a first extension announced with competitive bidding in 2023 to April 2025, and a further competitive extension announced in 2024 to March 2026. CFB followed on from Bristol’s Fulfilling Lives programme, known locally as Golden Key, which aimed to enable cross-sector collaboration and system change across multiple services to improve services for people facing multiple disadvantage [29, 30]. The local authority was awarded funding to establish the CFB programme and a multi-agency partnership, to test new ways of working in partnership. CFB brought three core themes to all its work: trauma-informed practice; equality, diversity and inclusion; and co-production with people with lived experience of MD. CFB delivery partner organisations included six voluntary and community sector (VCS) organisations who specialised in supporting people who have experienced multiple disadvantage, who were commissioned through a lead delivery partner (Second Step) to deliver the programme, with local authority adult social care having senior accountability. CFB programme staff, the local authority adult social care team and the six VCS delivery partners were invited to take part in this research through interviews and a staff survey. The National Health Service (NHS) Integrated Care Board and local NHS mental health trust were also CFB partners, but as they were less involved in programme delivery, they were outside the scope of this study.

### Study aims

This study evaluated the work of Changing Futures Bristol (CFB) and its partner organisations through the lens of TI practice. The aims reported in this article were to:

1. Analyse the enablers and barriers to trauma-informed practice across different services and explore how can these be overcome.
2. Analyse the impacts of implementing trauma-informed practice on staff, their organisations, and wider service systems.
3. Understand how trauma-informed practice can be linked across services, including wider system partners.

The study provides a longitudinal ‘deep dive’ into CFB, analysing TI activities and impacts within CFB as a programme itself and its wider influences and impacts across different partners. Further papers report on the implementation of trauma-informed practice within the lived experience organisation, Independent Futures (IF), that supported CFB in its co-production work [31], and the implementation of My Team Around Me, a co-produced intervention to provide co-ordinated support for clients of CFB (in production). The University of Bristol Faculty of Health Sciences Research Ethics Committee and local authority approved the research.

## Methods

### Participatory research approach

This evaluation used co-production approaches [32–35] to work in partnership with people through the lived experience organisation Independent Futures (IF) and CFB staff. The study was guided by participatory action research and co-operative inquiry where research is done with people rather than on them [36, 37]. Two people with lived experience of MD were involved in developing the evaluation funding bid and research design. To maintain confidentiality, it was agreed that research data would be collected by University researchers only, and only the University research team would have access to the data and conduct the initial analysis before sharing initial findings. In addition to the detailed research data collection procedures outlined below, the lead researcher (first author) attended Programme Board meetings as an observer, and was a member of the My Team Around Me evaluation group. This enabled the building up of longer-term relationships with key CFB staff and partners, and a stronger, broader understanding of the key operational and strategic issues that CFB faced and how these were addressed through the programme.

### Staff survey design, data collection and analysis

Outcome measures are under-developed in TI practice [26]. We discussed existing trauma-informed system measures [26, 38–40] with eight CFB staff and three IF lived experience members to identify the most appropriate existing measures to use. From this we developed a survey to meet the programme’s needs, using existing survey measures where possible, alongside programme specific questions. Existing standardised survey questions included the Trauma-Informed Systems Change Instrument which had factorial validity and internal consistency [39], co-production audit questions (chosen by CFB staff and lived experience representatives but less tested for validity and reliability) [42], general well-being measured by the short Warwick-Edinburgh Mental Wellbeing Scale which has shown internal consistency, test-retest reliability, and construct validity [43] and working conditions drawn from the European Working Conditions Survey [44], which has been tested for validity [45]. We added free text boxes on these different dimensions to provide an avenue for collecting qualitative data that may not be captured via the interviews. New survey questions suggested by CFB staff related to equality and diversity, which were informed by existent surveys [41], and asking staff about their own lived experience, as CFB were promoting the importance of this within the workplace.

An invitation to take part in the survey, hosted by the University’s secure survey system REDCap [46], was sent to relevant staff from the CF Bristol programme, six VCS partners and local authority adult social care teams, by CF partner leads who held staff email lists (Dec 2022-Mar 2023 – Timepoint 1). We repeated the survey after one year to understand any changes within organisations (Timepoint 2). Researchers did not have access to these email lists, but we understand from CF partner leads that the survey would have reached approximately 1,000 staff at both timepoints. CFB partner leads were unable to distinguish which staff may have participated in CFB activities, which is why the survey was sent out so widely, so this may have had an adverse impact on our response rates. Staff who had not previously participated were invited to complete a survey at timepoint 2 (Dec 2023-Mar 2024). New participants in this second survey were included in the second timepoint for the analysis. This was due both to high staff turnover in CFB and organisational changes in the year between the surveys. This means that for the first timepoint (T1) we had 85 responses, and for the second timepoint (T2), we had 62 responses. Overall, 117 staff responded to the survey, with 30 staff responding at both timepoints from all organisations – a reduction of about two thirds of staff from some organisations. Other limitations are that whilst the survey was co-produced with several partners, this meant that it included questions on a range of areas which increased its length. Not all participants completed every section, as all questions were asked on a voluntary basis. This limited the statistical power of a paired sample, and thus it was not examined for this study. Further, due to the inclusion of new staff in the survey at T2, and the above described organisational shifts within CFB, the timepoint-based approach was taken. Taking this approach focuses on describing the larger system changes across time, rather than individual staff experiences, which are described in the qualitative interviews.

Staff responded from CFB, 5 VCS partners and the local authority, as detailed in Table 2. Responses were categorised into three similar groups for analysis purposes as some smaller VCS organisations gave less than ten responses. The three groups were CFB which was a newly formed programme team, VCS partners who were more established, and the local authority as statutory partner.

**Table 2:** Staff survey responses categorised according to organisational type

Organisation type Notation: Staff Survey Organisational group	Timepoint 1 responses	Timepoint 2 responses	Total number of participants
Local authority Quote notation (SSLA)	*n* = 34	*n* = 19	42
Changing Futures Bristol Quote notation (SSCFB)	*n* = 15	*n* = 10	21
VCS CFB partner Quote notation (SSVCS)	*n* = 35	*n* = 30	53
Total	85 (1 organisation response missing)	62	117

For organisational type, CFB membership was prioritised for those who had listed more than one organisation, one person gave no organisational affiliation

Data were analysed using Stata 17.0 and R4.3.1, and descriptive statistics were calculated. To test whether there were organisational or time point differences, we used ANOVA or paired t-tests as appropriate for continuous outcomes, and chi squared tests for categorical outcomes. We checked the distribution of the outcomes to ensure parametric tests were appropriate. We performed two sets of tests. One set of tests was to determine, overall, whether there were any differences between responses for all staff at all organisations between each time point, i.e. ‘is there any change for everyone through time?’. The other set was to see if there were any differences between the three organisational groupings within a given time point, i.e. ‘is there a difference between organisation type at a single point of time?’. We did not apply multiple testing correction despite having a variety of outcomes and hypotheses, as the aims of the survey were twofold: firstly, to help inform the programme itself, and secondly as an exploratory analysis to link with the qualitative component of this study. Multiple testing correction is considered inappropriate for exploratory analyses, and the results presented here from the survey should be considered descriptive rather than confirmatory [47].

### Staff interviews, data collection and analysis

Inclusion criteria for staff interviews included service co-ordinators who supported CFB clients, CFB staff and strategic roles, other staff involved in the My Team Around Me intervention as identified by CFB staff, and CFB leads including from the six VCS partner organisations. The study was introduced to staff teams at various face to face meetings. Participant information documents were handed to people at the meetings and emailed to a wider selection of key CFB staff and lead partners involved in CFB. Sampling was on a purposive, volunteering basis and included key CFB programme staff roles, at a range of levels through the organisations, to get a broad overview of experiences. Key VCS partners who were involved within the CFB programme were also invited. From 32 invitations to take part, 23 staff volunteered and everyone who volunteered to take part was interviewed. Lived experience members were also interviewed, which forms the basis of a separate paper [31]. Participants could choose either in person interviews in a private meeting room, or online/telephone interviews at a convenient date. Participants gave written/verbal (recorded for online/phone) consent before being interviewed, which included processes for withdrawal of data at a later date. The topic guide was tailored to each interviewee as they had specific roles within CFB and iteratively developed to reflect thoughts from preliminary analysis. A copy of the interview topic guide is available in Additional File 1.

Twenty-three staff interviews were carried out. Interviews lasted between 39 and 67 min and were audio recorded, transcribed, checked for accuracy, anonymised and imported into NVivo for coding. Interviews were coded through an initial deductive coding frame informed by the TI implementation domains (Table 1) [48], with further contextually specific analysis (e.g. about the My Team Around Me intervention) conducted inductively using thematic analysis [48]. This thematic analysis adopted both semantic analysis looking at surface level meanings, and latent analysis to explore underlying meanings [49] in relation to how trauma-informed approaches were understood and practiced, with underlying assumptions explored. Two interviews were double-coded by the first and third authors. The first author coded all staff interviews, with further discussions between co-authors as analysis progressed, which supported reflexivity within the analysis process. Anonymised analysis was shared to discuss initial findings with key CFB staff and IF lived experience members, which further aided reflexivity and interpretation of data. Reflexivity was further enhanced through the write-up process where co-authors contributed to, discussed and shaped the write up process. Numbers after quotes notate the interview participant speaking. A summary of interview participants is outlined in Table 3.

**Table 3:** Summary of interview participants

**Interviewee characteristics**	**Numbers**
Organisation	16 Changing Futures programme, 7 VCS or LA partners
Age range	24 – 61
Gender	7 male, 15 female (1 not given)
Ethnicity	White British, White European, Black British, White Other, African (numbers not included due to small numbers and potential identifiability)
Line management	13 with managerial responsibility, 10 without managerial role

### Participatory analysis and write-up

We used a trauma-informed co-production approach to involve people with lived experience of multiple disadvantage [50]. In addition to the two Independent Futures (IF) lived experience members who supported the funding and design of the research, a further IF member co-presented initial findings at two national academic conferences and joined the write-up group. The IF member write-up team met regularly over twelve months. Members’ involvement was supported by IF staff. The write-up team commented on each article section once the analysis was anonymised and reviewed and edited the full manuscript.

## Results

Results follow the 10 implementation domains of trauma-informed practice (as detailed in Table 1). Some implementation domains are conjoined to provide a more integrated analysis, and others are augmented with further details from the thematic analysis that brought out additional dimensions to those listed within the implementation domains. This includes:

- an emphasis on all management levels rather than just governance and leadership;
- a specific focus on equality, diversity and inclusion as an implementation domain as well as principle (under cultural, historic and gender issues within the SAMSHA framework Table 1).
- understanding staff lived experience of trauma, which sits between the implementation domains of engagement and involvement and workforce development.

### Governance, leadership and policy

Short-term, competitive funding left less time and resources to cultivate trauma-informed ways of working. The national framing of Changing Futures initially as a three-year programme (July 2021- March 2024) set up tensions at a local level. When writing the bid, there were large pressures to secure funding, and time limits to significantly draw on all relevant local experience. Wider policy contexts directly impacted the implementation of TI practices, particularly where austerity funding cuts had impacted service provision. There were high ambitions to engage clients who were furthest away from services, and who had some of the most complex needs, working with many partners, and aiming to create systems change:

“It’s just, there’s too much, it’s over-ambitious and I think that’s done with the best of intention.” (Interview 17).

There was a transition period from the predecessor Golden Key programme, with some staff continuity, and many new posts recruited. A new office was established, and new policies, procedures, and working models were created. However, some new staff reported that their roles were unclear, and that the fast pace of the program added considerable pressures:

“Working in a complex space, trying to deliver a linear thing to pace – you know, it’s putting everyone in ‘Fight-or-flight’ mode… once you’re in ‘Fight-or-flight,’ you’ve in defensive mode, you cannot do creative work.” (Interview 12).

Staff appreciated working alongside committed, skilled and experienced colleagues. However, time pressures meant that it could be difficult to involve staff in decision-making, which could lead to a lack of transparency and lack of psychological safety.

Focussing on positive outcomes at a governance and policy level, the programme enabled the setting up of a new multiple disadvantage strategy across Bristol, held by the local authority, that blueprinted an approach toward multiple disadvantage (MD), embedding a cross-sectoral, collaborative approach, co-produced with lived experience members of Independent Futures (IF). The Multiple Disadvantage Transformation Board was also set up, comprising senior level executives including Public Health, Probation, Police, NHS ICB leads, and other similar heads of service and the MD Commissioners network, building on the area’s multiple disadvantage strategy. Having a trauma-informed lead helped to build networks across organisations, and movement toward more trauma-informed practice and policy, developing training and webinars, sharing learning and resources:

“It [TI practice] is in our language and if we keep embedding it into systems, into all different levels of having these conversations, I do think we are moving forward” (Interview 11).

### Management and TI practice

Managing complex, competing demands around finances, targets and system pressures whilst enabling a safe, relational TI space was a challenge. Management behaviours could shape organisational culture, which could then shape conditions of possibility for wider changes. Within demanding contexts, line managers could act as a protector from multiple pressures, giving staff space to do their work: “The biggest influence is probably your line manager” [27]. However, from interviews and staff survey comments, in organisations where there were significant pressures from performance targets, finances or national requirements, this protective space could be harder to create:

“Constant reviews of service and budget cuts causes undue stress and pressure to deliver.” (Survey SSLA78).

A managerial style mainly focused on delivery, or of a hierarchical nature, could contradict with a more open style that held complexity, uncertainty and space for learning. Reactive (rather than reflective) behaviour as a response to immediate demands, could be perceived in negative ways:

“Sometimes it’s come like a criticism, so ‘this is not trauma-informed’ has come like a criticism and I kind of feel like I’m run off my feet so I’m like, okay, I probably hadn’t thought about that but I haven’t really had the space to think about it, so it’s not something I’m actively not doing, it’s more that I haven’t even thought about it.” (Interview 17).

This highlights how staff, particularly in line management positions, need to be aware of how their actions and behaviours may impact upon the creation of a psychological safe culture. Because of the multiple system pressures, it was understood that TI practices such as appropriate staff support, reflective practice and clinical supervision, needed to be available for all including leadership and management, to embed a trauma-informed culture throughout an organisation:

“Trauma-informed approaches and reflective practice, and clinical supervision – and all those things that support that work around trauma – are seen as ‘Nice-to-haves’ within commissioning… rather than seeing them as the deeply intrinsic thing they need to be… without recognising that, without them, those services are unsafe for everybody involved.” (Interview 14).

Those in leadership and management positions needed to hold what could feel like conflicting demands such as performance targets, contractual obligations and restricted finances, whilst at the same time embodying TI principles such as safety, trustworthiness and transparency, empowerment, and inclusivity. Transparency in decision-making and clarity of expectations could be essential to get the balance right between these elements of management.

### Financing and funding

The political dimensions of how financing was distributed, created tensions and questions. Two interviewees questioned the structure that CFB had adopted, suggesting that more funding to existing partner organisations could have enhanced ongoing support and upskilled partner organisations to a greater extent:

“It’s almost like we’ve become another service, but there were services that existed in the city already…. For me, it would have just been better to upskill other organisations.” (Interview 19).

For Changing Futures, the political dimensions of allocating £3.3 million within specific organisations involved in different ways, set up its own dynamics, in a resource-limited system:

“I think when Changing Futures started there was quite a lot of negativity. It’s quite an expensive programme. So people looked at us and go, well, you know, why are you spending £3 million on that when our services are being cut and I can kind of understand that, but it’s been about seeing how we can support services and what we can do and what our offer is.” (Interview 16).

Other CFB partners highlighted how the broader funding context still systematically under-funded community-based, smaller organisations. There were attempts to ease this through building strong relationships with partners, sharing learning and resources, and being a route to influence wider system change. However, this did not solve funding issues for smaller community-based organisations, particularly within under-represented communities. Whilst there were commitments to learn collectively from these problems, the issues of appropriately and fairly sharing resources were ongoing.

Looking more broadly across the system, CFB VCS partners were experiencing significant pressures that impacted staff’s ability to implement TI approaches, including major re-procurement processes, loss of funding leading to closure of services for women with complex needs and associated staff redundancies, and another partner experienced a long period of industrial action (related to pay). Statutory services had to undertake large transformation programmes and service redesign. Financial issues related to uncertainties and delays in commissioning processes had a major impact on organisational/service planning and retaining staff, alongside uncertainty for clients they supported. Financing was a huge issue which could have major implications for all within an organisation. The stress of holding this was apparent:

“Financial sustainability of the organisation is a huge burden to carry.” (Survey SS30)

Financing and commissioning often set the conditions of possibility within an organisation/system and clearly impacted power relations and potential inequities through the allocation of resources.

### Equality, diversity and inclusion

Whilst equality, diversity and inclusion (EDI) falls within a TI principle within the SAMHSA framework (Table 1 under cultural, historic and gender issues), our findings highlight the importance of active implementation of EDI through an intersectionality and structural inequalities lens. The CFB programme worked with 3 different cohorts of people experiencing MD: women who had experienced domestic violence/abuse; people with experience of long-term homelessness and mental ill-health; and young people who had experienced racial trauma and criminal justice involvement. Staff who worked across the cohorts highlighted how the needs “of the three cohorts are very different, and experiences are worlds apart” (Staff 13), illustrating the importance of an intersectional approach. For example, homelessness across the women’s and young people’s cohort was much less visible i.e. “sofa surfing” than street homelessness. Others highlighted that the specific needs of young people had not been understood, nor had smaller specialist organisations who were doing the ‘hands on, operational work’ been appropriately resourced. For example, some Black-led organisations had less capacity to take part in learning events, as all resources had to be focussed on service provision. One partner highlighted how they were supporting Black-led organisations through the sharing of key policies and training provision. However, problems related to sharing financial resources remained:

“I think that’s the frustration for a lot of small organisations is that we do feel kind of embattled financially, but also that we feel that we’re doing all the work and everyone else is having meetings about the work.” (Interview).

CFB staff and partners commented on how a particular focus on EDI had helped them understand in a new way how structural racism and everyday racist incidents could impact people. Whilst this was seen as vital work to develop, there was also an acknowledgement that EDI work could be harder to measure and that there was still a long way to go.

“There needs to be more research and more conversations around especially racial trauma and the impact that it has. I think what tends to happen is as soon as someone says, ‘Racial trauma’, they think it’s just an immediate thing, but there’s a lot of deep stuff, generational stuff and a lot of stuff that plays out in society.” (Interview 11).

Other changes included developing recruitment practices to ensure a greater diversity of staff, and developing anti-racist practice, including anti-racist/EDI working groups and a Black-led forum.

### Staff survey results on equality, diversity and inclusion

**Fig. 1:**
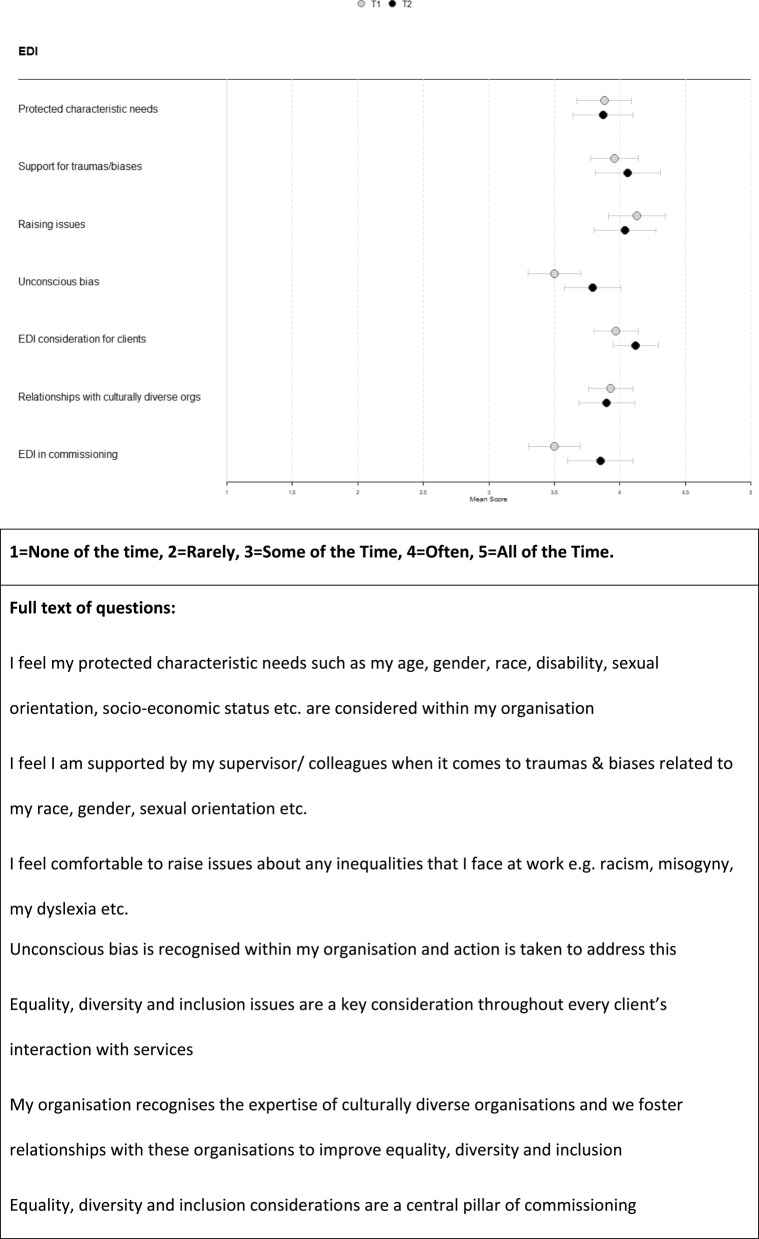
Equality, diversity and inclusion staff survey results^[1]^ [1] R code to make these coefficient plots can be found here: https://github.com/eyles-ec/useful-code-bits/tree/main/Coefficient%20plot

Figure 1 illustrates the results of staff survey EDI questions. One of the EDI items had organisational group differences. Organisational groups were significantly different on the item “Unconscious bias is recognised within my organisation and action is taken to address this,” at the second timepoint (T2 η² 0.052 p 0.044), but not the first (T1 η² 0.066, p 0.089). The lowest average score on unconscious bias in any of the time points for the organisations was 3.08 at T1 (just above ‘some of the time’ (3)) (CFB staff) and the highest was 4.04 at T2 (just above ‘often’ (4) (all other VCS organisations).

### Cross-sector collaboration

Within the CFB programme there were multiple cross-sector collaborative workstreams, including implementing co-production [31], equality, diversity and inclusion and trauma-informed practice, and wider systems change work for people experiencing multiple disadvantage, with specific leads in these areas. The Creative Solutions Board was a cross-sector collaborative forum which discussed how clients’ progress may be hampered by current systems, with an aim to develop longer term changes to prevent similar issues for other clients in the future e.g. problematic access to healthcare, housing issues. Whilst individual clients’ issues could be dealt with through flexing rules and boundaries, more long-term structural change was harder to initiate. Findings from the review of this Board included that professional relationships across services were key to enable client’s difficulties to be successfully tackled. The Creative Solutions Board developed into the setting up of Exchange Groups, with the System Stewards group which brought together managers and senior staff, and Practitioner Forums for operational staff, building partnerships to facilitate systems change, overseen by the Multiple Disadvantage Transformation Board. System change priorities included: support for families at risk of care proceedings following the difficult experiences of clients in this area, and how to best co-ordinate local support where prisoners were released early, due to a national shortage of prison placements.

#### Cross sector implementation of trauma-informed practice

The work of creating a trauma-informed environment and organisation takes time and space and it was acknowledged how CFB was beginning this process as a new programme, team and service. Not all staff were sure there was a shared understanding of what TI practice looked like through the programme or across organisations. Whilst there was a strong focus on TI practice in relation to clients, some interviewees recommended that there needed to be greater accounting of vicarious trauma and staff’s lived experience. Others spoke of the importance of opening spaces to navigate defensiveness in the context of limited resources and cuts in services to promote shared responsibilities across organisations. Cross-sector reflective practice was set up, partly to enable this. Interviewees who had participated in this highlighted how it strengthened peer support across organisations, but that further involvement of statutory services would also be beneficial. Other work that CFB supported was the facilitation of compassion circles to encourage self-care and compassion amongst colleagues, a cross-sector managers group to enable reflective practice across organisational boundaries and a learning collective to share good practice across the system.

#### Staff survey results on implementing trauma-informed practice

Some interviewees reflected that they considered that co-production and TI practice was more embedded within VCS organisations, in comparison with statutory services. These reflections were borne out by the higher scores on the co-production and TISCI scales when contrasting CFB, VCS partners and the statutory partner who took part in the survey, with the statutory partner having lower scores in these areas. These findings align with national evaluation results [51].

**Fig. 2:**
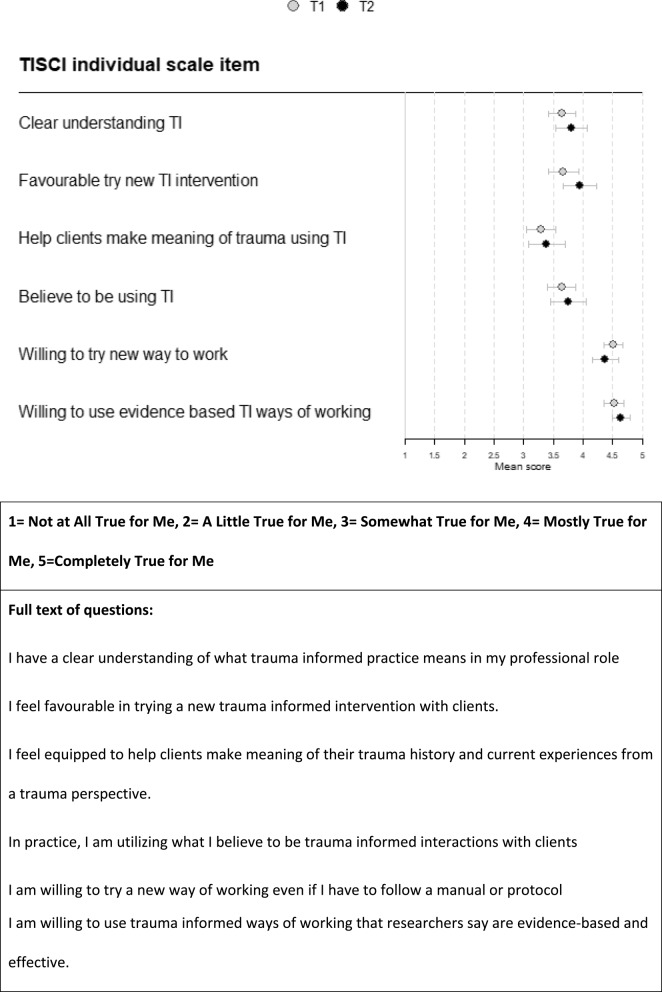
Trauma-informed system change instrument individual staff survey results

**Fig. 3:**
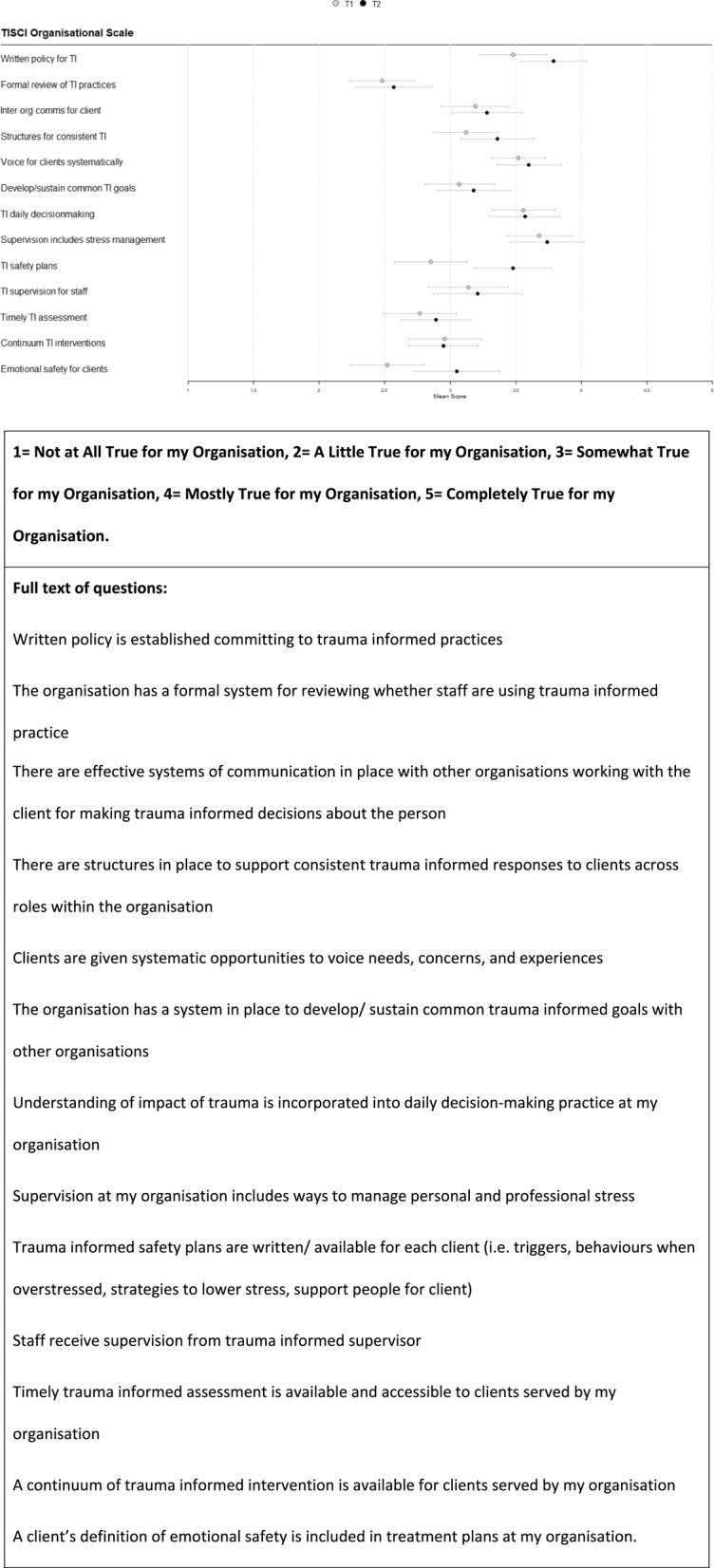
Trauma-informed system change instrument organisational staff survey results

Staff survey results of the TISCI are detailed below in Figs. 2 and 3.

Willingness to try new ways of working or trauma-informed ways of working were consistently highly scored across organisations (T1 mean 4.51 and 4.53 respectively in Fig. 2). In the case of clients’ definitions of emotional safety included within treatment plans, the mean score overall was rather low at the first timepoint (2.52) but improved over time (Fig. 3). At T2 VCS organisational groups and CFB improved by about one point to mostly true and became significantly different. This improvement aligns with the development of a CFB collective safety planning toolkit and the facilitation of shared safety plans across multi-disciplinary professionals who supported the same client through the intervention My Team Around Me (see Support services below). This may have had less impact on local authority staff whose work was dispersed over a much wider area. More generally between T1 and T2 there was an improvement on most TISCI scores at both an individual and organisational level, which aligns with all organisations’ attempts by CFB and other institutional initiatives to become more trauma-informed. However, the timescale of one year may have been too short for significant cultural changes to occur. There were no statistically significant differences between T1 and T2 when the scores from all the organisations were totalled, but movement was happening in a positive direction between the timepoints. Overall, the survey illustrated that individuals had a desire to implement more trauma-informed practice, but there were systemic issues that made it difficult to do this.

### Physical environment

Within the set-up of CFB, care was taken to ensure that the office space enabled a more TI environment. Some staff interviews highlighted an awareness of the importance of physical space in relation to: the physical environment where services were located; not wearing perfume to work as smells could potentially activate past trauma; and the difficulties within hostel spaces where some people may feel unsafe. There were overlaps with the equality, diversity and inclusion domain, particularly around the difficulties of disabled people in being able to access homeless hostel spaces, and overlaps with the finance domain, where finances were limited to re-design spaces to make them more trauma-informed.

### Engagement and involvement

Independent Futures (IF), the lived experience organisation funded through Changing Futures Bristol, carried out substantial work to promote involvement of people with lived experience of trauma throughout the Changing Futures programme and beyond [31]. The positive experiences of lived experience representatives within this team were different to the more mixed experiences of staff. Conscious enactment of TI principles by all, less pressure from delivery and complex systems change targets, line management, and staff involvement in decision-making appeared to enable a trauma-responsive and safe environment for the development, involvement and growth of lived experience members [31]. Our co-production survey responses are reported elsewhere [31], however more generally, resources for co-production were lacking through the wider system:

*“Current pressures on our service (long waiting lists and reduced staff numbers) means that we have little-no time for co-production.”* (Survey SSLA51).

Limited funding meant IF was unable to remain in its current membership form. Instead, CFB’s focus is now on embedding co-production and networking across existing lived experience groups.

### Staff lived experience and support

One interviewee expressed how there is a difference between lived experience representatives and staff with lived experience job titles.

*“One day they need to stop wearing the badge of lived experience*,* I hope*,* because you know*,* lots of us have lived experience in this team… I also really wanna make sure that it’s a way of people moving into employment either in the sector or elsewhere or just being able to move on in their lives.”* (Interview 17).

The prevalence of lived experience amongst staff was something we investigated within the staff survey. From 117 staff respondents across CFB and six of its partner organisations, 73% of staff reported that they had had direct lived experience of at least one domain of multiple disadvantage or had experienced trauma/adversity, with 55% reporting mental health issues, 14% reporting substance use issues, 9% reporting homelessness, 25% reporting domestic abuse, 7% reporting contact with the criminal justice system and 49% reporting experiences of trauma/adversity. 11% of staff had solely indirect lived experience through families/friends and 16% reported no direct or indirect lived experience. In interviews, some staff members shared aspects of their personal experiences, explaining how these experiences deeply motivated them to give their best effort in supporting clients:

“It’s personal… it means quite a lot to me. It’s not just a textbook I’ve read, it’s life experience”. (Interview 19)

Discussions about the high number of staff with personal experiences highlighted the differences between staff who may choose if to share any relevant lived experience, and staff that had lived experience within their job title. Those who have lived experience in a title may experience stigma, different pressures or expectations, in comparison with staff who have lived experience who may choose when and where they wish to share this. Some staff survey respondents felt very supported by their organisation, although others highlighted difficulties in the way they were managed, and one shared that their disabilities had not been fully accommodated. This highlighted the need for further work to ensure employment support and reasonable adjustments, improving human resource procedures to take better account of staff lived experience and disability rights.

### Training and workforce development

The number of survey respondents who had taken part in trauma-informed training stayed steady between timepoint 1 and 2, with 70% having taken part in training either facilitated by CFB or others at both timepoints. However, the numbers trained by CFB increased from 11 to 23%, whilst those trained by others decreased from 58 to 46%. Training resources may wax and wane in different parts of the system which contributed to these changes, highlighting the need for sustainable funding for TI training, especially where staff turnover is high. TI training that was led by both CFB and others was generally viewed positively by survey participants, which could affirm and connect with staff’s values around relational practice:

“I feel that being trauma-informed prioritises understanding, acceptance and emotional connection, which are strong foundations for relationship-based practice.” (Survey SSVCS07).

Further training and support for organisational leaders in TI practice was also seen as needed, as the position could be more isolating without so many peers. Managers highlighted their own needs for support when having to make difficult decisions and being able to step back to reflectively learn. One solution developed for this was to develop an externally facilitated senior leader TI reflective group.

Overall, more staff had taken part in reflective practice between timepoint 1 (68%) and timepoint 2 (77%), although VCS partner numbers involved in reflective practice dropped during this time (made up for by increases within LA and CFB staff). Where reflective practice existed, it was generally well-received. Group reflective practice helped people to learn from peers, individual reflective practice enabled issues to be raised where there might be discomfort in a group setting or with peers. However, reflective practice could mirror wider pressures felt by staff, so it was important not to perceive this as a cure-all:

“Quite honestly a lot of frustrations that the staff go to reflective practice with is completely out of our control and is down to the commissioners and higher up.” (Survey SSVCS106).

One CFB partner had set up a consultation working group where staff could vote to discuss issues in a forum with leaders. Whilst some of the issues brought up were not easily solvable, others did initiate change and it was considered that the “power of acknowledgement” (Interview 29) in this was vital. Greater staff involvement and transparency in decision-making could counteract some of the difficulties that staff reported, reducing tendencies to a hierarchical approach with more collaborative management.

Vicarious trauma and staff well-being were issues highlighted across staff interviews and survey. Working with highly distressed clients could have a significant impact on support workers. Some spoke of difficulties where clients were directly abusive to staff, and whilst they recognised this was a trauma-response from clients, the emotional impact was hard to carry. The pressures of direct work with clients could be intense, especially where there was less support for clients from statutory services at a time of crisis e.g. mental health:

Accessibility is a huge issue. A lot of my clients seem to fall through the cracks of every single mental health service that is on offer in ((City)), of which there are not many…. It does lead to quite a lot of vicarious trauma because then it is just me.” (Interview 21).

This could have a significant impact on staff wellbeing and turnover. Across partners, the substantial costs of burnout were highlighted as a key issue that needed to be addressed:

“Staff wellbeing is a massive funding issue, because every time you have to recruit, it costs you thousands of pounds to go through a recruitment process” (Interview 26).

The sense of feeling undervalued as a frontline support worker was apparent.

### Support /treatment services

A key intervention developed by CFB was My Team Around Me, which was a collaborative multi-agency strengths-based approach for working with people experiencing multiple disadvantage. Each CFB client had their MTAM managed through a service co-ordinator role, who was seconded to CFB from each of the six VCS CFB partners. Key MTAM principles were to be client led, with shared accountability and service continuity, and innovative practice through person-centred trauma-informed approaches. Service co-ordinators managed and co-ordinated multiple relationships, with the client and range of services that they needed to access (which could include housing, probation services, health services, welfare benefits, social worker etc.):

“People with multiple disadvantage have lots of people [practitioners] involved and if they are all doing different things or they’re siloed or they’re not communicating, that’s just replicating the problems in the system.” (Interview 32).

Where MTAM succeeded, benefits included:

- time and efficiency savings that could avoid work duplication across agencies,
- more transparency on what work was happening across services and what could be done differently,
- more reliable and up-to-date information on clients,
- professional peer-support to temper compassion fatigue and burnout,
- stronger co-ordination between services.

Appropriate communication channels across different organisations were a key facilitator to this, and whilst IT system barriers existed, simple solutions in the form of shared secure anonymised email communications became workarounds. There were multiple challenges to this way of working including variations across organisations in their referral criteria, different treatment plans, limited timing to access services, continuity of care and named professionals.

### Evaluation, monitoring and quality assurance

Our findings illustrate that trauma-informed evaluation methods need further development and implementation. All CFB client beneficiaries needed to complete CF nationally designed evaluation questionnaires at the beginning of their support and at three-month intervals. However, the phrasing and length of these questionnaires was often problematic, and questions could be very sensitive and personal, which had implications for their ongoing usage.

“They can’t sit and go through questions and some, a couple would find it very, very triggering.” (Interview 21).

These time limited, short-term evaluations did not take account of the long-term relational approach that may be needed to work with people who may have experienced trauma over decades, and have long term multiple disadvantage:

“The clients that we are working with have a huge trauma and a lot of overlying complexity on that. On average, I think 15 years. The clients that I’m working with, the complexity, homelessness, mental health, drug use, existed more than 10 years, 15 years. Imagine someone who is 40 years old, 20 years in a very chaotic lifestyle, when you ask him about his young age, (-) possibly he was abused. That reminds him, abuse from parents or I don’t know, whatever. And that really triggers anger. I have seen it. I have to stop sometimes, they get angry with me and I said, ‘Sorry, this is not about me. It’s about the questionnaire.‘” (Interview 22).

The national client evaluation forms were challenging to complete in a trauma-informed way, and service co-ordinators highlighted triggering or culturally inappropriate questions, alongside response choices that did not fit the situations of CFB clients. This type of evaluation can drive a hierarchical and transactional approach, rather than one that was trauma-informed.

To summarise, Table 4 highlights key findings in relation to the first research question to understand the enablers and barriers to implementation of TI approaches, how barriers can be overcome and implications for action.

**Table 4:** Interview findings and evidence-based implications/ recommendations

Implementation Domains [4] and Findings	Evidence-based implications/ recommendations (from issues raised in main text and table)
Policy Within the development of CFB there were multiple and far-reaching ambitions, which could be difficult to match in practice due to the complexity of systems change. Wider policy contexts directly impacted the implementation of TI practices: *“The impact of frontline working with marginalised people in the context of 10+ years of austerity, dismantling of public services, and the normalisation of extreme poverty is severe levels of burnout and vicarious trauma, and this feels like it getting steadily worse. The only things that are going to repair this are the restoration of the welfare state, reforming the criminal justice system and drugs policy, and properly funding existing frontline services - rather than throwing millions at new 'learning projects' and initiatives.”* (Survey SSVCS27)	Localised programmes are always situated within national policies and contexts, which shape what is possible at a local level. The adverse impacts of austerity on services cannot easily be mitigated at a local level. Competitive funding processes can fuel over-reaching ambitions, that are difficult to then realise in practice, under-acknowledging and building on existing good practice. Avoid “reinventing the wheel” (Interview 24). Trustworthiness and transparency are important principles to maintain when developing policy and strategy (what is likely to be possible given the context). Focus action on levers that are possible to influence and most favourable to change. Transparency and greater acknowledgement of constraints and difficulties that may impede progress may be helpful.
Governance, Leadership & Management Leaders and line managers had an important influence on psychological safety and transparency in decision-making. This was impacted by finances, funding requirements and policy pressures: “*Often, our systems, and our processes, and our organisations mirror the trauma in the individuals we support! …. Even where that’s unconsciously… it’s about trying, always, to take that step back and have a broader lens on that stuff, and seeing how those things ripple all the way through*.” (Interview 14) Governance and leadership actions and behaviours could shape cultural contexts, which could then shape conditions of possibility for wider changes. Line management was an important buffer to create space for TI practice: *“If you have a line manager who in a monthly supervision is reflective, is supportive, is trauma focused and informed and is thinking about your wellbeing and the risk of vicarious trauma, I think that's the biggest influencer of you adopting that sort of approach as a practitioner”.* (Interview 27) *“I think one of the biggest barriers is senior strategic leads, expecting other people to change their mindset and not willing to reflect themselves”* (Interview 26) Parallel processes, where trauma and relationship dynamics are repeated through different levels of an organisation [67] need to be consciously and compassionately reflected upon.	Managers and leaders must hold contradicting drives to deliver targets and manage finances whilst creating space for relational support and TI practice. Finding a balance with all these elements is important. Appropriate support for leaders who hold multiple, complex demands is as important as front-line staff support and supervision. All staff need to be aware of how their actions and behaviours impact others, particularly in line management positions with a view to creating a culture of psychological safety. Recognising emotions that can lead to a reactive response (hyper/hypo arousal) is important to counter unconscious or less reflective behaviours. To appropriately attend to parallel processes [67], where difficult interactions may be repeated, all staff, particularly leaders, may need to attend to practices to enhance self-awareness (observing one’s own patterns of behaviour), self-regulation (monitoring one’s own emotional responses) and manage their connection with others [53].
Financing and funding Greater consideration for how funding and commissioning can build value and support small community-based organisations is important. CFB partners found that annual commissioning cycles for services were particularly challenging to work within: *“It's really destabilising for them as a team, and takes focus away from what needs to be delivered, and takes focus away from being innovative, because your headspace is taken up with other things really.”* (Interview 28)	When developing funding bids, consider how inequities in funding can be addressed between organisations. How can smaller community-based organisations be given more resources to be able to support their work? How can commissioning support more participatory, community-led and collaborative practice, that focusses more on values and relationships [64]? Short-term commissioning creates an uncertain context which can lead to higher staff turnover and distraction from support work. How can longer-term commissioning be encouraged?
Equality, diversity and inclusion Understanding intersectionality within diverse populations who experience multiple disadvantage is particularly important to account for people’s different needs when accessing services. A greater acknowledgement of structural racism and understanding of the impact of racial trauma needs to be embedded as part of a TI approach: “*I think [racial trauma] needs to be embedded more into the framework and into people’s language about recognising the different levels of racial trauma and the impact of that on people*” (Interview 11).	When working in the area of multiple disadvantage be aware of how different cultural, gender and historic issues may affect access to services. Inequities in funding distribution can mirror wider inequities in the system. Further work to understand the impact of racial trauma is important, acknowledging how racism can impact individual experiences and ongoing structural inequities that need to be challenged and changed.
Cross sector collaboration Cross sector collaboration was vital to share resources and navigate support for clients. Enablers included: - building professional relationships across services. - opening up spaces for learning, to navigate defensiveness and promote shared responsibilities across organisations/ sectors: *“Everyone’s resources are being cut but it’s why it’s even more important that we’re working together and sharing those resources and supporting each other.*” (Interview 16). - larger organisations sharing resources with smaller ones, including training, access to reflective practice, policies. Barriers to collaboration included: - multiple other pressures on services: *“We're still wanting to work closely in partnership, but the ability to think in a partnership first way hasn't been straightforward.”* (Interview 31) - cultural, financial, professional practice and referral differences between statutory and VCS organisations. - large caseloads in other service organisations made it difficult to collaboratively engage, due to limited time.	The sharing of key policies, the provision of training across organisations, cross-sector reflective practice and learning events, all enabled wider learning across partners. Capacity funding like Changing Futures is needed to facilitate and build this cross-sector collaboration, especially where service demands are high. However, funding to be able to attend these events may also be needed, especially for smaller organisations that are only paid for their service provision, with little capacity building support. Understanding the constraints and demands of all organisations is very important. Identifying key people who understand the need and will actively champion collaboration is essential.
Physical environment Office spaces were designed to ensure a more TI environment. There was an acknowledgement that other buildings such as hostel environments can be traumatising for both clients and staff, especially where there is no private space, but that resources to re-design spaces are limited. There was problematic access to housing for people with physical disability who were experiencing homelessness.	Physical environments can be harder to change and may need substantial resources to re-design them, to become more trauma-informed. Agencies should carry out a psychologically informed environment audit as a matter of course.
Engagement & involvement Sufficient resourcing, support and embodiment of TI principles were vital to enable a safe space for lived experience representatives to contribute [31]. Most staff had experience of at least one domain of multiple disadvantage or adversity/ trauma. Staff had different perspectives on human resources support, particularly for people with lived experience, and some survey respondents saw that further HR work was needed in this area, related to disability policies.	Resourcing, support and embedding TI principles can create a safe space for people with lived experience to get involved and have voice within organisations and systems. For co-production to be meaningful, all levels of governance need to be more participatory [31]. Lived experience of staff is likely to be under-accounted for. Further acknowledgement of this and staff support in this area is important. Human resources departments need to consider the large number of staff who may have their own lived experience, and what additional support might be necessary for staff, to counter vicarious trauma, burnout, and the re-triggering of trauma. Human resources departments need to consider their statutory requirements around disabilities and support to staff. Demarcating and labelling specific roles as “lived experience” or “peer” may detract from embedding a cultural norm of encompassing lived experience [59].
Training & workforce development Vicarious trauma was very apparent through the staff team, with a sense of a lack of support around this which contributed to staff turnover: *“If you have a high turnover you are just throwing money away, so why not spend the money on actually valuing staff, lowering caseloads. I am sure I am not saying things that commissioners haven’t thought about a million times, but I just feel really strongly that that’s an important thing to continue addressing.”* (Interview 26) Staff involvement in decision making was seen as needed, but not always apparent in practice: *“A lot of the time, staffs feelings, ways of working, concerns, policy input etc about certain topics is never ever noted - all client focused, we don’t get heard - THAT ISNT CO-PRODUCTION!” *(Survey SSVCS106). Reflective practice, whilst helpful, could mirror wider pressures in the system: *“I do get monthly reflective practice which there isn’t a lot of reflecting done, it’s more about venting feelings or at least they’re really emotionally charged which perhaps indicates that there is lack of support overall.”* (Interview 24)	Staff wellbeing is a huge issue that costs organisations through burnout, staff absence and turnover. There is a need for a greater awareness of staff vicarious and direct experience of trauma. Staff need appropriate support and acknowledgement of the difficulties of the work. More staff involvement in decision-making may help staff feel valued, and support trust and transparency. Managers should be clear about what and how staff will be involved in decision making. Where multi-disciplinary teams and secondments are in place, thought needs to be given as to how staff are supported by both their home and seconded organisation. Whilst reflective practice is very important, it is not a cure all and wider pressures in the system can be felt most acutely by front line staff.
Support/ treatment services My Team Around Me could provide integrated and co-ordinated services for clients, but there was variation in the extent to which different services and sectors engaged with this, partly dependent on caseloads, referral criteria and capacity. Different organisational criteria limit the opportunity for consistent, named professionals to be involved in integrated and co-ordinated services.	Cross sector collaboration is vital to provide integrated support for people with MD, but challenging, as different organisations vary in their referral criteria, treatment plans, limited timing to access services, continuity of care and named professionals.
Evaluation, monitoring & quality assurance Nationally designed evaluation questionnaires were not seen as trauma-informed, and included triggering questions about past and present trauma. They were seen as ‘*service-led’* rather than ‘*client-led’* (Interview 15): *“They're not always a priority for that client at the time... Some days they don't even want to talk to you… my male clients, a lot of them are quite involved in criminal exploitation, so they’re pulled in all different directions all the time. I can call up and say, ‘I've got this lovely £20 voucher, come and do a survey with me,’ but they're getting paid £300 to sit in a trap house, I can’t compete with that time.”* (Interview 19) It was seen that different ways of evaluating and measuring services needed to be developed to greater account for the relational, values base of this work: *“‘Do we need to think about totally different ways of approaching the way that we lay out specifications?’ ‘Should we be measuring different things?’ If we’re looking at things from a truly trauma-informed lens, actually, you know, ‘Are there different approaches to the commissioning process that could be explored?’*” (Interview 14)	Surveys for clients who’ve experienced multiple disadvantage and complex trauma, need to be co-designed with people with lived experience so that they mitigate any potential re-triggering. Funders and commissioners of services who support people with complex trauma need to consider alternative approaches to commissioning and evaluation that take account of the relational, values-based nature of the work. Outcomes, outputs and throughputs do not measure the extent to which organisations are trauma-informed. To embed these TI approaches, more work needs to be done at a commissioning and contracting level, to understand how to best to monitor progress in this field. How can commissioning be framed to enable a greater valuing of community-led, relational practice [64]?

## Discussion

This study aimed to understand how Changing Futures Bristol implemented trauma-informed approaches in areas related to multiple disadvantage including (a) the enablers and how barriers can be overcome (b) the impacts of implementing trauma-informed approaches and (c) how trauma-informed approaches can be linked across services and system partners. Table 4 has outlined facilitators and barriers across the 10 implementation domains [4].

### Enablers and overcoming barriers to TI approaches

Enablers included:

- governance and leadership prioritising this at all levels including development of the local multiple disadvantage strategy,
- additional resources for collaborative working and lived experience input,
- support for cultural change to integrate a trauma-informed approach into existing policies and practices,
- support for leaders to practice more TI approaches,
- cross-sector collaboration and reflective practice,
- seeing that a TI approach was something that all staff needed to work towards (including HR and finance teams),
- TI training for skills development.

Training courses need sustainable funding, especially where staff turnover is high, to embed TI cultures. Resources and literature in TI leadership are emerging [52–54], and there is a need for further research in this area [52]. Practically, managers may need to consider attending to team dynamics [52]; their own practices to enhance self-awareness, self-regulation and connection with others [53]; attending to emotional and relational team pressures to ensure that they are contained to maintain psychological safety [52, 54]; and increasing awareness of and critical challenge to incorporate diversity and inclusion into trauma-informed leadership [55].

Managers and leaders had to manage contradicting drivers to deliver targets within tight budgets whilst creating space for relational support and TI practice. It is essential to provide adequate support for managing these conflicting demands, just as it is important to support and supervise front-line staff. Building trust and being transparent are crucial values that benefit both staff and clients, helping to create a psychologically safe environment.

Achieving improvements in TI approaches is an ambitious goal that can be hampered by institutional and system pressures, barriers including short-term competitive funding and commissioning cycles. Nationally, CF faced these difficulties over several areas, and this can be a challenge as long-term systems change can be incremental over a longer period [24]. Short-term funding can also lead to difficulties in staff retention, due to short-term contracts which in turn can lead to high staff turnover [25]. Other stressors can compound this including vicarious trauma, stress and the pressures of the job, lower than average salaries and challenging working conditions.

Broader contextual challenges included the continuing austerity and large funding gaps for local authority services, which has led to serious challenges in balancing budgets [56]. The implementation of co-production and budgets for involvement of people with lived experience was hampered by lack of resource, as illustrated in the staff survey. Within wider system partners, the closure of services for women with multiple disadvantage, hit the very people who experienced the highest health inequities [57]. Other barriers cited included national issues related to the criminal justice system and drugs policy, and the need to “properly fund existing frontline services”. These issues continue to highlight how austerity can impact those who are most vulnerable [58]. Short-term funded programmes cannot easily mitigate these wider impacts of austerity and broader structural inequities.

### Impacts of implementing trauma-informed approaches

To answer the second research question on the impacts of implementing TI practice, a slow move toward more TI cultures within organisations could be detected through the staff survey responses after one year, although this did not reach levels of statistical significance. A one-year period may not be sufficient to evidence cultural change across a range of organisations, when many other cost drivers and re-structuring across services were also happening. That individual and organisational TISCI scores showed positive movement after a year illustrates a perception amongst survey respondents that initiatives to become more trauma-informed, both by CFB and other partners, were having some effects. However, limited short-term funding for continuing TI initiatives and lived experience involvement can threaten the embedding of this, even though strategic ambitions existed. Full resourcing, support and the embodiment of TI principles by everyone can create a safe space for people with lived experience to get involved and have voice within organisations and systems [31]. However, lived experience of staff is less visible. This study highlights the large numbers of staff (73%) who self-identify as having experienced at least one domain of multiple disadvantage or experienced trauma/adversity. Lived experience is widespread, and some staff mentioned that this was a key reason they were motivated to work in this sector. Our findings align with the Groundswell report [59] on stigma within lived experience roles, that recommends that everyone should have a choice in disclosing personal experiences, and that lived experience can be understood as a cultural norm (which this study illustrates that it is) rather than the exception [59]. Given the high levels of personal experiences within the workforce, this finding highlights: (a) the question of whether it is helpful to have specific “lived experience” roles in a job title, or whether this can also be stigmatising; (b) the need for extended trauma-informed human resource support for staff, to ensure well-being and career progression [59]. Appropriate support for all staff is important to avoid stigmatisation, potential re-triggering and vicarious trauma. Human resources departments need to consider their statutory requirements around disabilities and support to staff in this area. This study aligns with the national evaluation findings that workforce turnover can adversely impact the embedding of TI practice, and that staff support is needed at all levels of the organisation [51]. Staff well-being is a vital area to focus on to avoid burnout, absence and turnover. Ensuring staff involvement in decision-making may help staff feel valued and motivated, and support trust and transparency.

### Linking trauma-informed approaches across services and system partners

To answer the third question on linking TI approaches across the system, a long-term, integrative, collaborative and trauma-informed approach is needed at all levels, including leaders, managers, policy and central government, to overcome key barriers. Capacity funding like Changing Futures is needed to facilitate and build cross-sector collaboration, especially where service demands are high and, in some cases, increasing. Cross sector collaboration is vital to provide integrated support for people with MD, but challenging, as organisations vary in their referral criteria, treatment plans, limited timing to access services, continuity of care and named professionals. However, this collaboration needs to be more long-term and embedded into existent institutional structures. It has been highlighted by national CF partners that the nature of competitive bidding means that programmes may be over-ambitious and focus on the new rather than improving the quality and co-ordination of existing provision, which can reduce value of progress made [24].

Consistent action and partnership working is needed to tackle issues related to equality, diversity and inclusion, and specifically racial trauma and inequities, using an intersectional approach. Racial trauma is inter-generational and societal. Funding patterns could mirror wider structural inequities where smaller community-led organisations had less resource. This mirrors evidence from the United States that highlights how racial biases can affect philanthropic and grant-making processes in inequitable ways [60]. This highlights the continuing need to challenge structural racism and inequities, so that changes can be made to structures that perpetuate inequities.

Nationally work is needed to understand how greater local autonomy can be reconciled with traditions of centralised accountability [61]. This was most visible through the national evaluation questionnaires which had the potential to re-traumatise clients through their in-depth personal questions. Trauma-informed approaches to measurements are developing [62] and evaluations for clients who’ve experienced multiple disadvantage and complex trauma, need to be co-designed with people with lived experience so that they mitigate any potential re-triggering of trauma. Strategies that encompass both trauma-informed and output/outcome goals across all levels of the system are needed, in order for transparency, trust and accountability to be visible across the system [63]. Funders and commissioners of services who support people with complex trauma need to consider alternative approaches to commissioning and evaluation that take account of the relational, values-based nature of the work. Local authority mission-led procurement that encourages more participatory, community-led and collaborative commissioning, and that focusses on values and relationships offers a way forward [64].

This local cross-sector collaborative commissioning needs to be mirrored at a national level with collaboration across government departments and national policy, ensuring work on the various dimensions of multiple disadvantage is better co-ordinated. This includes establishing cross-departmental collaboration to ensure polices and funding related to multiple disadvantage is integrated, longer-term and flexible to local area needs [24, 25]. This aligns with a mission-led approach to government, where long-term objectives are set to tackle complex challenges which require cross departmental and cross-sector collaboration [65]. Intrinsic motivation of staff was apparent and they are already a “coalition of the willing” [65], but the conditions in which staff work needs to support their nurturing and growth. This means replacing duplicative, competitive short-term funding with larger dedicated funding streams that means local services can plan for the longer term, and that staff have more stability in their roles and build on existing good practice [24, 25]. Trauma-informed approaches are closely aligned with new models of public service reform that value a ‘high trust, high skill, high autonomy’ workforce, where power is devolved and services become more personalised [66].

### Strengths and limitations

The strengths of this study are that it was able to longitudinally follow the development of CFB, conducting an in-depth study of how the programme developed, and its impact on wider partners. It was co-produced from the beginning to the end with lived experience representatives being involved in the research funding bid, research design, analysis and write up. Data includes interviews with key CFB staff and partner organisations and the survey reached a far wider range of Bristol partner stakeholders than the national evaluation (only 2 Bristol partners completing the national partners survey (Table A1.10 [51]) in comparison with 95 staff from Bristol partner organisations completing our survey). Our analysis illustrates that to fully implement TI approaches, as well as the 10 implementation domains already detailed by the SAMHSA framework [4], further implementation work is explicitly needed at all managerial levels, not just governance and leadership, and that equality, diversity and inclusion needs to be incorporated as an additional implementation domain, to be able to tackle intersectional inequalities.

Limitations are that whilst the survey was co-produced with several partners, this meant that it included questions on a range of areas which increased its length. Not all participants completed every section. This means that the statistical power of the survey was limited not only by this but also recruitment issues due to staff turnover. Thus the survey results should be considered descriptive as it is exploratory analysis, which enhances the narrative of this study. Further, due to those organisational changes it was difficult to recruit a paired sample, thus staff were invited to complete a survey at T2 who had not previously completed a survey at T1. This means that that some variation will be introduced as participants may not have answered both surveys, and the power of a paired sample is lost. However, the survey enabled us to understand the wider movement toward TI practice across several partners. Finally, as we did pool the partner organisations together for the timepoint analysis, it could account for the difficulty in discerning statistically significant effects. It suggests that similar surveys should be conducted across partners and other services in other areas to perform confirmatory analysis. The wider systems movement toward TI practice across a broader range of partners is currently being further evaluated. Other limitations include that this article focuses solely on staff perspectives. Lived experience representatives are fully detailed in a separate article [31]. Service users’ perspectives were evaluated through qualitative interviews conducted by a separate University, and national evaluation questionnaires, thus we were careful not to replicate existing work.

## Conclusion

Multiple disadvantage by its nature is related to deep structural and social inequities, which can be challenging to address through short-term funded programmes. People who have MD often have experienced deep and sustained trauma. Having a TI approach is essential to build longer term trusting relationships with clients, and for support staff to be able to work consistently and for the longer term. Staff turnover loses that trust and relationship, for people who may have been let down multiple times and have attachment issues. For staff to be trauma-informed, the system also needs to provide better working terms and conditions for staff, with appropriate leadership support, throughout the system. To conclude, Table 5 outlines what this means at different systems levels in practice.

**Table 5:** System level changes to support more trauma-informed practice within multiple disadvantage

System level	Key actions
National policy level	Build broader national cross-sector collaboration to ensure both policies and funding on the various dimensions of multiple disadvantage is better co-ordinated [24]. Use mission-led government principles and public service reform models that prioritise long-term approaches, the devolution of power, trust and autonomy in the workforce [65, 66]. Provide longer-term financial settlements so that local services can plan ahead, build on existing good practice and reduce reliance on short term commissioning of services [24]. This will in turn enable longer term staff contracts and may reduce staff turnover. Accountability and outcome measures need to be designed with support staff and clients who will complete them, to ensure that they are accessible, reliable and not asking questions that may re-trigger trauma.
Local funding and commissioning	Focus on different procurement models that encourage more community-led and collaborative commissioning, that builds on a relational, strengths-based approach as well as delivery and accountability [64]. Ensure that funding allocations tackle structural inequities to allocate community-led organisations more resources to tackle intersectional inequalities. Ensure longer term funding for TI training so that all staff can benefit from this and embed it into regular training plans so that it is part of established practice. Commissioners should fund protected supervision hours to mitigate vicarious trauma.
Leadership and management practice	Provide reflective space and external support for managers so that they can attend to conflicting system demands, team dynamics, and emotional and relational pressures when working with trauma, to ensure psychological safety for all. Increase awareness of how to incorporate diversity and inclusion into trauma-informed leadership [55]. Encourage more participatory practices where possible. Ensure a culture of trustworthiness and transparency, alongside psychological safety for all staff [52]. At the same time, this needs to strike a balance with openness to critique and challenge to encourage best practice. Focus on staff well-being to avoid burnout, absence and turnover. Be mindful of the likely presence of lived experience within the workforce.
Human resource management practice	Ensure human resource support is more available for staff to support disability rights, vicarious trauma, and the complex emotional dynamics that can arise when supporting people who have experienced complex trauma. Consider whether lived experience in job titles is necessary given the high levels of personal traumatic experience within the workforce generally.
Research and theory	Develop further resources and research to understand how leaders can promote more trauma-informed cultures within contextual constraints. Further research is needed to understand how intersectional inequalities may affect the diverse populations who may experience multiple disadvantage. Including equality, diversity and inclusion as an implementation domain within trauma-informed approaches may support greater awareness of intersectional inequalities within TI implementation. Whilst integrated care teams such as My Team Around Me showed promise, further research is needed to understand how they can be embedded sustainably within services where there are different system demands.

## Supplementary Information

Supplementary Material 1.

## Data Availability

Data associated with this manuscript is accessible only to the research team and is not publicly available due to concerns about confidentiality in a study based in identifiable organisations, with a specific locality and small sample size.

## Abbreviations

CFB: Changing Futures Bristol
MD: Multiple Disadvantage
MTAM: My Team Around Me
SAMHSA: Substance Abuse and Mental Health Services Administration
TI: Trauma-informed
VCS: Voluntary and community sector
