# Supplementary material for: Implementing trauma-informed practice across services to support people experiencing multiple disadvantage: a mixed method study
Source: BMC Health Serv Res. 2025 Oct 1;25:1266. doi: 10.1186/s12913-025-13339-8 (PMC12487007; doi:10.1186/s12913-025-13339-8)
Supplement: Supplementary file 1 — Supplementary Material 1. [file 12913_2025_13339_MOESM1_ESM.docx]

# Changing Futures Bristol: Trauma Informed Evaluation

*Initial staff interviews topic guide*

1. **Can you tell me a bit about your job role, responsibilities, how you are involved in Changing Futures? Are you happy to share with me your gender, age range, and ethnicity?**
2. **What’s your professional background? What motivates you personally in your role?**
3. **Can you tell me a bit about how your role has developed since you’ve started with Changing Futures?**
4. **How have you gone about developing the trauma informed (TI) TI work?**
   1. What are the key issues that you’ve been focussing on?
   2. Enablers/ barriers to the work

The core ambitions of Changing Futures are to embed equality diversity and inclusion, co-production and trauma-informed practice into services and systems, so the following questions start to explore these:

1. **What does a good trauma-informed approach look like in practice to you?**
   1. **What are the important characteristics of a trauma-informed approach at a organisational/ system level? How can we link the practitioner level with the strategic level?**
   2. **What do you see as the key local barriers that you hope to tackle through Changing Futures? How?**
   3. **What are the wider national/policy barriers to this?**
   4. **Is there anything that you see that could be developed within Changing Futures to encourage more trauma-informed practices?**
2. **How does a trauma-informed/ co-production approach sit within Changing Futures when there are also significant delivery pressures to reach funders targets?**
3. **Have you been involving lived experience representatives/ Independent Futures (IF) members in your work? Can you tell me how you’ve gone about doing this?**
   1. What’s happened through this process?
   2. How did you support IF members to get involved? Were there any specific barriers? What, if anything, helped?
   3. How effective did you think their contribution was? Why?
   4. How were hierarchies managed within this? (prompts: delegation, deadlines)
   5. Have there been examples of where people have disagreed or had differences in values or conflict within a co-production approach? How have these been managed?
4. **How is equality, diversity and inclusion being promoted through your area of work?**
   1. **Are you able to work at a systems level in this area? If so, how?**
   2. Have you been able to support/ take part in conversations about race/ gender/ unconscious biases/ other equity concerns?
   3. How did that go?
5. **Have you been working with commissioners at all? Can you tell me more about that and what that involves?**
6. **What do you need to be able to embed co-production, TI practice and equality diversity and inclusions (EDI) into system wide work?**
7. **Moving onto longer term system outcomes, can you share how your work might link with the longer-term system change ambitions:**
   1. **Are you involved in shared safety planning and if so how?**
      1. How is risk-taking being supported in your role? What has happened? Can you give examples?
      2. **How might shared safety planning work in practice across organisations?**
      3. **Enablers to the work?**
      4. **Barriers and how these can be overcome Prompts: different organisational practices/ needs, technology**
   2. **Does your role involve influencing commissioning processes at all? If so how?**
      1. Enablers to the work?
      2. Barriers to integrating trauma-informed approaches/ EDI/ co-production into commissioning processes? How can these be overcome?
   3. **What do you think are the key conditions and/or mechanism that need to be created for longer-lasting cultural and system change so that people facing multiple disadvantage (MD) can live a life beyond services?**
      1. Enablers to the work?
      2. Barriers and how these can be overcome?
   4. **How does your role contribute to the My Team Around Me (MTAM) approach?**
      1. What are the key issues that need to be tackled to facilitate mainstreaming of MTAM?
      2. Enablers to the work?
      3. Barriers/ biggest challenges and how these can be overcome?
8. **Where can you go for supervision and support?**
   1. Is there anything that is currently happening that makes you feel unsupported? How would you like that to change?
9. **What do you hope that Changing Futures will have achieved by the end of the programme in March 2024?**
10. **What do you think that this trauma-informed evaluation needs to focus on over the next year? What advice would you give me? Are there any particular colleagues/ partners that you would advise that I also speak with?**
11. Is there anything else you’d like to add before we finish?

*questions for interviews with service co-ordinators:*

1. Can you tell me a bit about your job role, responsibilities, how you are involved in Changing Futures? Are you happy to share with me your gender, age range, and ethnicity?
2. What’s your professional background? What motivates you personally in your role?
3. How are you approaching sessions with clients? Can you share a bit about your relationships with clients and how you’ve developed these? Where people have engaged less, why do you think that is and how have you tried to approach this?
4. How is My Team Around Me working in practice? Do you think it is different from your usual way of practice? If so how, if not why not?
5. How are relationships developed with different agencies and professionals through MTAM? Do they see MTAM as a different way of working? If so how? If not, why do you think not?
   1. Have you worked with specific MTAM delivery tools? (Prompts: meetings/ formulation/ shared safety planning/ used EDI tools)
   2. What are the enablers and barriers to this work?
   3. Are there any particular agencies or professionals outside of the CF partners that you think would be useful for me to talk to about their experiences and perceptions of MTAM?
   4. Through your work have you asked people within the system to flex to take account of clients’ needs? What happened?
   5. Do you feel able to make any lasting change? If so how, if not – why? (Prompts: policy/ strategy/ system change)
6. What do you need to be able to do your job as well as possible?
   1. Where can you go for supervision and support?
   2. Is there anything that is currently happening that makes you feel unsupported? How would you like that to change?
7. How is equality, diversity and inclusion considered within clients interaction with services?
   1. Is EDI related trauma recognised by different professional groups within MTAM?
8. How can MTAM be scaled up?
   1. Do different organisations prioritise MTAM clients’ needs differently? Explore prioritisation of clients across organisations and different system demands
   2. How do you see that shared safety planning might work in practice across organisations?
   3. Where do responsibility and accountability lie within a MTAM approach?
9. Are their specific issues that need to be taking into account with the group of people that you are supporting? i.e.
   1. Young people
   2. Women experiencing domestic abuse
   3. People experiencing complex/compound trauma, chronic homelessness
10. Have you asked any clients to complete the national evaluation survey questions?
    1. How have you approached this?
    2. What have clients responses been?
    3. Have there been any adverse reactions to any particular questions?

*CF Partner interviews*

1. Can you tell me a bit about your job role, responsibilities. Would you be happy to share with me your age range, gender and ethnicity – these would only be reported as a total to describe the range of respondents?
2. How are you are involved in Changing Futures?
   1. How has your work connected with Changing Futures through the programme?
3. Can you tell me about your hopes/ expectations of the Changing Futures programme before it began?
   1. Were you involved in the planning and development of the programme?
4. How does your organisation work with/ support the service co-ordinator who has been seconded from your organisation? What has that arrangement been like? Is there anything you’d want to see developed in these arrangements?
5. Do you think that the My Team Around Me model that the service co-ordinator is involved in developing is an approach that could be adopted within your own organisation? Why/ why not?
   1. CF are also piloting how shared safety planning can work in practice. Have you had any experiences of this? What’s your perspective on how this might work?
6. Have you connected with the CF trauma-informed lead, systems change and EDI lead? How has that worked? (Prompts to expand as necessary)
7. What does a good trauma-informed approach look like in practice to you?
   1. What are the important characteristics of a trauma-informed approach at a organisational/ system level?
   2. What do you see as the key local barriers? How might they be tackled?
   3. How do you think commissioners might be involved in nurturing a trauma-informed culture?
8. Are you doing any work in your own organisation about promoting more trauma-informed practice? Can you tell me about that?
   1. What barriers have you come across in this work? (Prompts: national/ local/ structural barriers):
9. What are the key things that you need within your organisation to support the embedding of:
   1. more trauma-informed practice?
   2. more co-production?
   3. more equality, diversity and inclusion?
10. Have you been involved in other parts of Changing Futures? Prompts:
    1. Involvement in Creative Solutions Board/ delivery group/ cross-sector reflective practice/ Programme Board/ other CF meetings?
11. We’ve been conducting the trauma-informed staff survey that your organisation was part of. Across all partner organisations, 73% of staff shared that they had some personal lived experience. Does that result surprise you? Would you consider there are any implications of this for staff support?
    1. Do you have any processes to support vicarious trauma for staff? How does this work?
12. Through the time of CF different partners have had other pressures such as funding cuts/ strikes.
    1. Have there been particular issues that you’ve had to manage?
    2. Have these impacted your work with Changing Futures?
13. What benefits has your organisation experienced from being a partner within the Changing Futures programme?
    1. What would you like to see change in the system as a result of the CF work?
    2. Are there any resources/ toolkits/ products that would be helpful for CF to produce?
    3. Key learnings from the work with CF?
14. What advice would you give Changing Futures to help plan the extension of the service co-ordinator work and the work of Independent Futures for another year?
15. What do you think that this trauma-informed evaluation needs to focus on? What advice would you give me? Are there any particular colleagues/ partners that you would advise that I also speak with?
16. Is there anything else you’d like to add before we finish?
